# A first evaluation of the efficacy of minibeam radiation therapy combined with an immune check point inhibitor in a model of glioma-bearing rats

**DOI:** 10.1016/j.ctro.2025.100911

**Published:** 2025-01-06

**Authors:** Lorea Iturri, Emmanuel Jouglar, Cristèle Gilbert, Julie Espenon, Marjorie Juchaux, Yolanda Prezado

**Affiliations:** a Institut Curie, Université PSL, CNRS UMR3347, Inserm U1021, Signalisation Radiobiologie et Cancer, 91400 Orsay, France; b Université Paris-Saclay, CNRS UMR3347, Inserm U1021, Signalisation Radiobiologie et Cancer, 91400 Orsay, France; c Institut Curie, PSL Research University, Department of Radiation Oncology - Paris and Orsay Protontherapy Centre, F-75005 Paris, France; d New Approaches in Radiotherapy Lab, Center for Research in Molecular Medicine and Chronic Diseases (CIMUS), Instituto de Investigación Sanitaria de Santiago de Compostela (IDIS), University of Santiago de Compostela, A Coruña, Spain; e Oportunius Program, Galician Agency of Innovation (GAIN), Xunta de Galicia, A Coruña, Spain

## Abstract

Glioblastoma multiforme (GBM) continues to be a hopeless case today. Its treatment involves the use of multiple modalities. One of them is radiation therapy (RT), that is limited by normal tissue tolerances in GBM patients. GBM is widely recognized to induce local and systemic immunosuppression, which is a hindrance to the use of immune-modulating therapies. One possible strategy is to ally immunotherapy (IT) with novel RT approaches able to revert the immunosuppressive nature of GBM. One example is minibeam radiation therapy (MBRT). Preclinical experiments have shown that MBRT leads to a remarkable widening of the therapeutic window for GBM and elicits an effective immune priming. The main hypothesis of this study is that the activation of the immune system by MBRT would synergize with IT enhancing tumour control and minimizing toxicities. To validate it, *in vivo* experiments in a glioma rat model were performed. The goal was to assess the gain in survival of animals treated with MBRT, MBRT plus an immune check point inhibitor (ICI) versus conventional RT (CRT) or ICI alone. All treatments (ICI alone, CRT, CRT + ICI, MBRT and MBRT + ICI) increased survival with respect to the non-irradiated controls. However, the high radiation dose (30 Gy) delivered in one fraction in CRT is highly detrimental for normal tissues contrary to MBRT. The combination of CRT plus ICI appeared to be toxic. MBRT + ICI surpassed the survival rate with respect to ICI alone. When ICI was used with high-dose conventional irradiation, tumor eradication was observed in 6 out 8 animals. However, the survival was statistically equivalent to MBRT plus ICI, with a tendency to reduce survival in comparison with CRT alone, suggesting acute toxicity associated with this multimodal treatment.

In conclusion, our results suggest some advantages for MBRT in combination with ICI. We need to conduct further work to determine the optimal RT-IT combination and schedule for ICI injection and MBRT irradiation.

## Highlights

• Glioblastoma multiforme (GBM) continues to be a hopeless case today.
• One possible strategy is to employ novel RT approaches able to revert the immunosuppressive nature of GBM.
• We hypothesize that the immune activation by MBRT would synergize with IT enhancing tumor control and minimizing toxicities.
• All treatments (immune checkpoint inhibitors (ICI) alone, CRT, CRT + ICI, MBRT and MBRT + ICI) increased survival.
• The use of ICI combined with high dose conventional irradiation resulted in some toxicity, not observed with MBRT.

## Introduction

Glioblastoma multiforme (GBM) ranks among the most lethal forms of human cancer [1]. No effective treatment exists yet today. Radiation therapy (RT) has a key role in solid tumours’ treatments (50 % of patients in the EU) [2]. Normal tissue tolerances compromise its effectiveness in treating GBM and some other radio-resistant bulky tumors [3].

Immunotherapy (IT) holds promise to revolutionize the field of oncology [3], with adoptive cell transfer and checkpoint blockade being the main strategies employed in the clinics.Its clinical success in solid tumors has, in many cases, been limited due to many different barriers [4], such as the irregular stroma and vasculature of those tumors [5], immune suppressive cytokines and suppressor cells and T cell exhaustion. The low tumor mutational burden (TMB) and the immunosuppressive microenvironment of GBM make it hard for immune effector cells to get inside the tumor. This makes the response to immunotherapy (IT) lower and the chance of immune checkpoint inhibitors (ICI) working less likely. However, there is emerging positive clinical evidence in medulloblastomas, tumors that share these unfavorable features [6]. Moreover, no association between higher TMB and improved survival in GBM could be demonstrated [7]. Randomized phase III GBM clinical trials with an immune check point inhibitor (nivolumab) showed no survival improvements [8]. Limited antitumor response was also observed using CAR-T therapy in three different clinical trials [9], [10], [11]. A novel strategy, p32-CAR T cells [12], has shown to be able to recognize and eliminate not only glioma cells but also tumor endothelial cells. The alliance of IT with other therapeutic strategies that can reverse the immunosuppressive factors could be a game changer. Accordingly, the immunomodulatory role of RT is influenced by the immune context of cancer, the total dose, the dose per fraction, the method of dose delivery, and the duration of the treatment. While the optimal effective doses and fractionation for immune priming remain elusive [13], it is widely recognized that conventional fractionation schemes (2 Gy per fraction over several weeks) usually create an immunosuppressive microenvironment, and hypofractionation shifts the balance towards immune stimulation [14]. But the high risk of toxicity that comes with hypofractionation in some large radioresistant tumors, like GBM, makes it less useful in conventional RT (CRT).

New RT approaches based on distinct dose delivery methods can potentially lead to differential (favorable) immune effects while minimizing the risk of toxicities, even in hypofractionation regimes. This is the case of minibeam radiation therapy (MBRT) [15], [16]. MBRT uses an array of sub-millimetric beams resulting in highly heterogenous dose distributions. A significant reduction in normal tissue toxicities [17], [18], [19], [20], [21] was observed with X-rays and proton MBRT. Significant increase in life span was observed in glioma bearing rats with X-rays MBRT [22], [23], [24]. An equivalent or even superior tumour control (up to 83 % GBM eradiation) than in CRT have been reported after proton MBRT in small animal experiments. [25] The two first MBRT patients with advanced tumours (Merkel cell carcinoma and recurrent preauricular squamous cell carcinoma) have been treated at Mayo clinic Rochester USA [26]. MBRT led to symptoms relieve and shrinkage of the tumour with no toxicity for the time of follow-up.

Classical radiobiology concepts cannot explain MBRT [27]. Among the differential proposed mechanisms [16], immunomodulatory effects are likely to play the major role. A crucial role of T cells in the antitumor response of MBRT has been demonstrated [24], [28]. MBRT led to a faster and more efficient immune tumour infiltration, dominated by cytotoxic and helper T cells at the centre of the tumour. This was in contrast to homogeneous CRT, where T cells were blocked around the edges of the tumor [24]. MBRT has thus shown an immunomodulatory potential by improving effector immune cell accessibility to the tumour, either through specific inflammatory pathway activation or modulation of the extracellular matrix. Moreover, MBRT can lead to the establishment of a long-term anti-tumour immune response [24]. Finally, MBRT also increases monocyte tumour infiltration [24]. Those play an essential role in secreting type I interferon in the TME [29] and the RT-induced CD8^+^ T cell anti-tumour effect [30] correlated with the efficacy of immune checkpoint inhibitors (ICI) and RT combinations in GBM [6].

The immunomodulatory effects of MBRT along with the neurotoxicity reduction encourage the investigation of MBRT-IT combinations. We hypothesise that: i) ICI can positively influence the immune infiltration driven by MBRT by blocking T cell exhaustion, leading to an enhanced anti-tumour response, and ii) ICI and MBRT combined can further increase glioma survival.

To verify those hypotheses, we have performed an exploratory *in vivo* study in a rat glioma model. To the best of our knowledge, this is the first evaluation of the combination of MBRT and ICI.

**Fig. 1:**
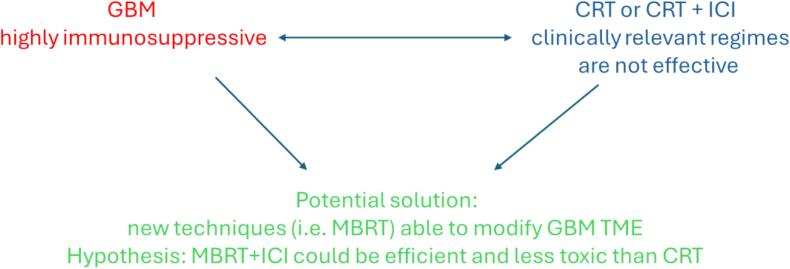
The problem statement and the main hypothesis of the study.

Fig. 1 summarises the problem statement and main hypothesis of this study.

## Materials and methods

Ethical statement: All animal experiments were conducted following our institutions' animal welfare and ethical guidelines and were approved by the Ministry of Research (permit no. APAFIS #44683–2023091322031874 v1). Animals were housed at the Institut Curie animal facility accredited by the French Ministry of Agriculture for performing experiments on rodents. Cages were enriched with cardboard tunnels.

### Tumour inoculation

We used the transfected RG2-[D74] (ATCC® CRL-2433TM, RRID: CVCL_3581) glioma cell line with the luciferase gene and GFP reporter gene (RG2-Luc-GFP).

We mixed 50,000 RG2-Luc cells with 5 µL of DMEM and injected them into the brains of wild-type Fischer F344 rats (Janvier Labs, France) that were 6 weeks old. We used a Hamilton syringe and a burr hole in the right caudate nucleus (which was −1 mm front to back, +4 mm side to side, and −5.5 mm dorsal to ventral from the skull). Before irradiation, bioluminescence imaging (BLI) confirmed the presence of a tumour. BLI was conducted using an IVIS spectrum system (Perkin Elmer, Houten, The Netherlands). We injected D-luciferin intraperitoneally at a concentration of 150 mg/kg and measured bioluminescence with the IVIS spectrum 25 min later (peak of bioluminescence). Only rats displaying a BLI signal significantly exceeding the background signal on the day prior to irradiation were enrolled in the study. We performed group randomisation based on BLI signals to ensure that each group exhibited a similar average BLI signal. The clinical status of the animals during the experiment was checked five times per week. We humanely euthanised any rat exhibiting classical adverse neurological signs associated with tumour growth or substantial weight loss using CO_2_ asphyxia.

### Irradiations and immune check point inhibitors injections

Unilateral X-ray MBRT and CRT were applied using a small animal irradiator, as previously described [17]. A tension of 220  kV and an intensity of 13  mA were employed with inherent and additional filtrations of 0.8  mm and 0.15  mm of Beryllium and Copper, respectively. This results in an energy spectrum with an effective energy of 69  keV. The irradiation area was 1.2 cm x 1.2 cm in both MBRT and CRT. The average dose rates were 4.2 Gy/min and 1.3 Gy/min (3.6 Gy/min in the peaks) for CRT and MBRT, respectively.

Dose delivered to the tumor was calculated with Monte Carlo (MC) simulations (TOPAS v3.2). Our MC dose engine was validated by means of experimental measurements with films in a solid water phantom [17]. After that, MC simulations were performed in computer tomography images of a rat head to assess the calibration factor between the dose in the solid water and the dose in the rat brain for the irradiation field used (1.2 cm x 1.2 cm).

**Fig. 2:**
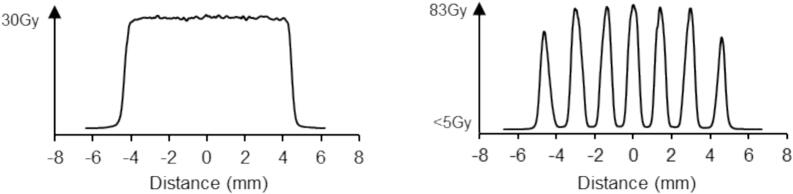
Lateral dose profiles at the tumor position for CRT (left) and MBRT (right).

A therapeutic dose in our tumour model (30 Gy) was prescribed [24]. Previous studies showed that 25–30 Gy mean doses are needed in both conventional and MBRT irradiations to a have a significant increase of lifespan with some long-term survivals in the glioma-rat model employed [24]. In MBRT, the delivered peak and valley doses were 83 ± 7 Gy and 4.5 ± 0.3 Gy, respectively. The mean dose in MBRT is defined as the average dose between the first and last peaks of the lateral dose profile. Fig. 2 shows the lateral dose profiles at the tumor position, where the beam width was 700 ± 40 µm, and the center-to-center (c-t-c) distance between minibeams was 1400 ± 100 µm.

Irradiation was carried out 14 days after tumour inoculation. From previous studies employing the same model, a tumour volume of 60 ± 22 mm^3^ on average, with an average axial dimension around 5.5 ± 1.0 mm is to be expected the day of the irradiation. Radiochromic films were then placed on the skin for quality assurance of the irradiation.

**Fig. 3:**
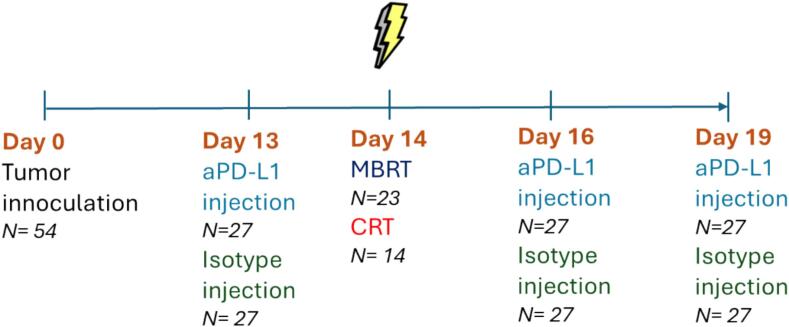
Timeline of the experimental procedures.

Every 3 days starting from the day before the irradiation (−1, 2, 5 days post-irradiation [31], [32]), we injected part of the animals intraperitoneally with either anti-programmed death-ligand 1 (anti-PD-L1, 10 mg/kg BioXCell) or IgG1 isotype control (10 mg/kg BioXCell). Table 1 shows the groups included in the study. The study was performed in three independent experiments containing all the groups in each experiment. Fig. 3 shows the timeline of the experimental procedures.

**Table 1:** Groups distribution.

	Dose	Number of animals
Non-irradiated controls + isotype control	0 Gy	9
Non-irradiated controls + aPD-L1	0 Gy	8
CRT + isotype control	30 ± 1 Gy	7
CRT + aPD-L1	30 ± 1 Gy	7
MBRT + isotype control	30 ± 2 Gy, 83 ± 7 Gy (peak) and 4.5 ± 0.3 Gy (valley)	11
MBRT + aPD-L1		12

### Histology

Animals were humanely euthanised by CO_2_ asphyxia. The whole brain was harvested and immersed in zinc-formalin-fixative (Z2902-3.75L, Sigma-Aldrich). After fixation, the tissues were embedded in paraffin and microtome sectioned at a thickness of 6 μm. Serial sections were used for haematoxylin-eosin saffron (HES) staining for histopathology evaluations. The HES images were acquired in BFx1 using a Nikon AZ100 Multizoom microscope.

## Results

**Fig. 4:**
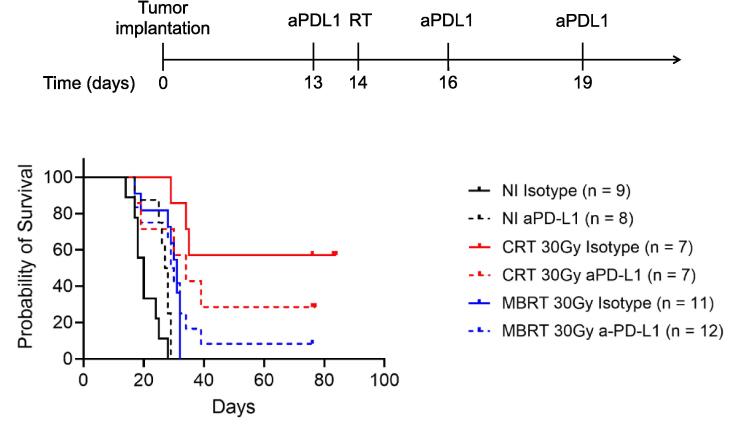
Upper panel: the experimental plan followed in the study. Lower panel: Survival curves of RG2-bearing rats in all of the conditions tested. NI refers to non-irradiated animals, CRT to conventionally irradiated animals, and MBRT to minibeam radiation therapy.

Fig. 4 displays the survival curves of rats with glioma following treatment with ICI, RT, or a combination of RT and ICI. Employing a PD-L1 immune checkpoint inhibitor (non-irradiated (NI) aPD-L1) results in a statistically significant (albeit modest) increase in survival (p = 0.01) compared to the controls (NI isotype). Furthermore, all irradiated groups experience a significant increase in survival when compared to the controls.

**Fig. 5:**
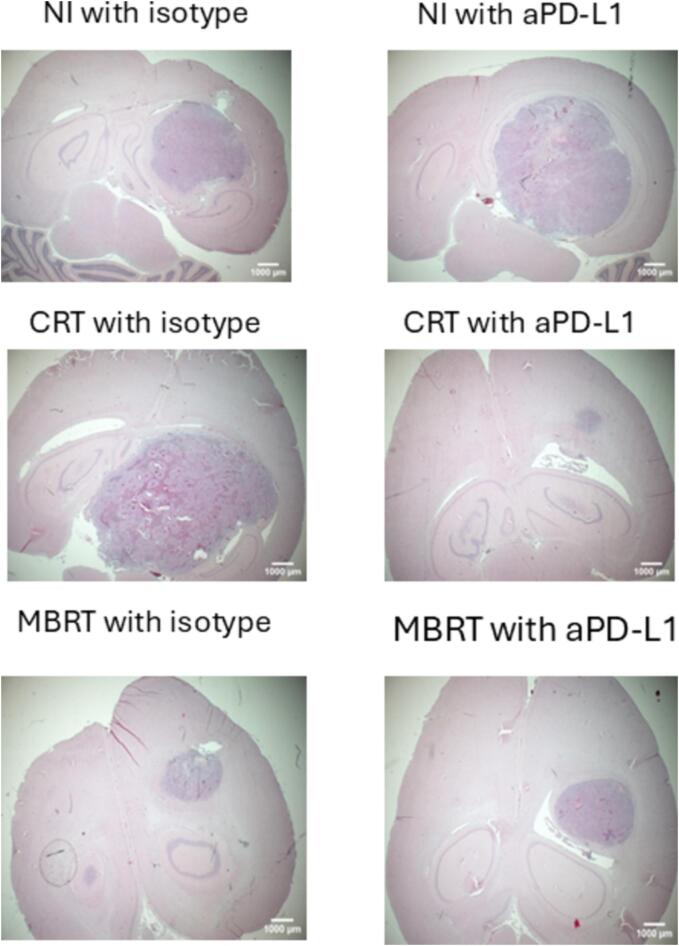
Representative HES images of animals in the different study groups.

MBRT alone significantly increases the survival with respect to ICI alone (p = 0.008) although the combination of both MBRT + ICI does not improve survival with respect to MBRT alone. After MBRT + ICI, one animal achieved long-term survival free of tumor. The best survival results are obtained when CRT is employed alone. However, this treatment (30 Gy in one fraction), on the other hand, is not a treatment option because it is too harmful to healthy tissues in the medium and long term [24]. Interestingly, the combination of CRT with aPD-L1 was statistically equivalent to MBRT plus aPD-L1 and it showed a tendency to reduce survival in comparison with CRT alone, although not statistically significant (p = 0.2561). Indeed, the HES revealed that in 6 out 8 animals of this group the tumour was eradicated and in the other two animals the remaining tumours were every small. Therefore, the animals’ death was not due to tumour growth but to toxicity of the combined treatment. Tumour was also absent in the animals surviving the length of the study (3 months, N = 3) in the conventional irradiation group plus isotype and in the MBRT plus ICI group (N = 1), while the other four animals died from tumour growth. Tumour presence was observed in all other animals of other groups. Fig. 5 shows some representative HES images of the different treatment groups.

## Discussion

GBM remains an urgent medical need. The current standard of care, which involves gross total resection if possible, followed by radiation therapy (RT), concurrent chemotherapy (temozolomide, TMZ), and maintenance TMZ +/- tumour treating fields [1], [33], results in a modest median progression-free survival [34] of 4–6 months. It is also linked to toxicity and sequelae in the central nervous system, which can lower quality of life, especially for the growing number of patients who have been alive for more than two years.

IT is an emerging cancer treatment [3]. However, achieving clinical success with IT for solid tumours has often proven challenging due to various barriers [4]. These barriers include the tumors' irregular stroma and vasculature, which can promote hypoxic and acidic conditions. These conditions create a challenging environment for immunotherapy performance, leading to immunosuppressive cytokines and suppressor cells, as well as T cell exhaustion. Therefore, combining immunotherapy with therapeutic strategies that can counteract immunosuppressive factors could significantly improve the outcome.

The reduction of neurotoxicity along with the effective immune response provided by MBRT made us hypothesize that the alliance of MBRT and ICI could be a game-changer for GBM. With that aim, we performed a first exploratory study. All treatments increase survival with respect to the controls. The most important increase in increase lifespan was achieved when high dose CRT was employed alone. However, the important toxicity observed with this dose regime [17], [24] prevents its clinical use in contrast with the reduced toxicity in MBRT [17], [24]. The use of ICI combined with high dose in CRT was very efficient in tumour eradication (75 % of the animals). However, the animals died early, with a shorter lifespan that the animals receiving conventional RT alone. This suggests acute toxicity of this combined treatment, no observed in the MBRT groups, and probably linked to systemic uncontrolled inflammation. Moreover, CRT plus aPD-L1 did not increase the survival with respect to the MBRT plus aPD-L1. MBRT alone increases the survival with respect to ICI alone. Potential explanations are the need for a higher valley dose (4.5 Gy was employed in this study), or the fact that treatments were delivered at a very advanced stage of the tumour. As the tumour grows, there is a larger necrotic core, and less structured vasculature. Both factors might hinder the ICI action both in terms of accessibility and presence of infiltrated T cells. Thus, an earlier injection of ICI might increase efficacy. Linked to that, it might be that the immune priming in MBRT is more related to vascular impact than in CRT and the fact of having a necrotic core was more influential in MBRT than in CRT.

It should be noted that in this study we have compared CRT and MBRT in terms of mean dose, as in previous studies [24], [25], [35]. There is an intense debate in the community about the optimal way to compare both irradiation modes [16]. Some other works suggest employing equivalent uniform dose (EUD) based on in vitro cell survival [36]. However, EUD models currently used are based on the linear-quadratic model, which assumes radiation-induced clonogenic cell death is only affected by the radiation dose the cell receives. None of the main biological mechanisms of MBRT such as bystander effects, abscopal effects, vascular effects, and immune modulation are taken into account by using EUD.

Even though our results suggest that the toxicity reduction in MBRT as compared with CRT, further work is needed to find the optimal treatment time and delays between ICI injection and MBRT irradiation. The temporal scheme that we have used was inspired by previous successful preclinical studies on radio-immunotherapy combinations using the PD1/PD-L1 axis blockade [31], [32], but it might not be suitable for the case of GBM and MBRT. The most recent clinical trials suggest that PD-1 blockage treatment (nivolumab) might not be suitable for [37] and could be worse than current standard temozolomide treatment [38]. The investigation of the use of another type of ICI, like those targeting CTLA4, CD25 or immune-suppressive inflammatory pathways is warranted, as they could better synergize with heterogeneous dose distributions in central nervous system malignancies. In addition, some additional studies targeting less necrotic and extracranial tumours (thus, no potential negative impact of the blood–brain barrier) would be of interest.

## Conclusions

The combination of CRT and aPD-PL1 was able to eliminate the tumours in 75 % of the animals, but it led to acute toxicity and reduced lifespan as compared with CRT alone. MBRT can reduce toxicity, and both MBRT and MBRT and aPD-PL1 enhanced survival with respect to non-irradiated controls.

Therefore, further research is required to determine the most effective IT and MBRT combination strategy for a successful and long-term treatment.

## Declaration of competing interest

The authors declare that they have no known competing financial interests or personal relationships that could have appeared to influence the work reported in this paper.
